# Identification of Contractions from Electrohysterography for Prediction of Prolonged Labor

**DOI:** 10.2478/joeb-2022-0002

**Published:** 2022-03-31

**Authors:** Santosh N Vasist, Parvati Bhat, Shrutin Ulman, Harishchandra Hebbar

**Affiliations:** 1 Manipal School of Information Sciences (MSOIS), Manipal Academy of Higher Education (MAHE), Manipal, India; 2 Melaka Manipal Medical College (MMMC), Manipal Academy of Higher Education (MAHE), Manipal, India; 3 Philips Healthcare, Bangalore, India

**Keywords:** uterine contractions, prolonged labor, fundal dominance, electrohysterography

## Abstract

The analysis of the uterine electrical activity and its propagation patterns could potentially predict the risk of prolonged/arrested progress of labor. In our study, the Electrohysterography (EHG) signals of 83 participants in labor at around 3-4 cm of cervical dilatation, were recorded for about 30 minutes each. These signals were analyzed for predicting prolonged labor. Out of the 83 participants, 70 participants had normal progress of labor and delivered vaginally. The remaining 13 participants had prolonged/ arrested progress of labor and had to deliver through a cesarean section. In this paper, we propose an algorithm to identify contractions from the acquired EHG signals based on the energy of the signals. The role of contraction consistency and fundal dominance was evaluated for impact on progress of the labor. As per our study, the correlation of contractions was higher in case of normal progress of labor. We also observed that the upper uterine segment was dominant in cases with prolonged/arrested progress of labor.

## Introduction

According to WHO, there were 2.6 million stillbirths in 2015, around 7000 newborn deaths every day in 2016, and approximately 810 women died every day from preventable causes related to pregnancy and childbirth in 2017. From 1990 to 2017, a quarter of all women who died during childbirth had undergone a cesarean section [1]. The number of cesarean section deliveries globally was estimated to be 22.9 million in 2012 [1]. The rate of cesarean section deliveries in India in 2015-16 was 17.2% [1]. Prolonged labor is one of the major reasons for non-elective cesarean section deliveries at 11.65% [1]. Cesarean sections can be lifesaving in conditions such as dystocia, but unnecessary cesarean sections pose a high risk to safe outcomes and add to the global healthcare expenditure.

The progress of labor is said to be prolonged when the labor duration exceeds 20 hours in primipara and 14 hours in multipara women. The prolonged labor may occur due to abnormality in uterine contraction, size of the baby, or shape or size of the pelvic passage, of which uterine contractions are the only unpredictable factor. Timely identification of prolonged labor and intervention (cesarean section) could reduce mortality and morbidity associated with it. Access to skilled care at low resource settings and geologically isolated areas has been limited due to workforce shortage [1]. Higher care centers are facing the challenge of planning and budgeting of staff required to provide safe and effective care [1]. This may lead to unit congestion, inappropriate use of beds, increased length of stay, long waiting times and patient dissatisfaction, increased hospital expenditure, inefficient use of staff, and staff dissatisfaction [1].

Studies have shown that monitoring of the electrical activity of the uterine muscle through Electromyography (EMG), sometimes also referred to as Electrohysterography (EHG), to be better than Cardiotocography (CTG) in monitoring the uterine contractile activity [1]. The uterine

contractions are commenced by the electrical activity in the myometrial cells, due to the de-polarization and repolarization of the smooth muscle cells [1]. Although a number of studies [10, 11, 12, 13] have attempted to predict prolonged labor based on the EHG patterns, the outcome is not clear and convincing.

## Materials and methods

The objective of this study was twofold; one was to identify the contractions from the EHG signals. The other more important objective is to evaluate the role of contraction consistency and fundal dominance in the progress of labor.

**Fig.1:**
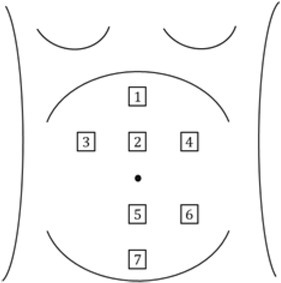
Representation of surface electrode positioning on the abdominal surface.

Uterine activity during the active phase of labor, i.e., 3-4 cm of cervical dilatation was recorded using Electro-hysterography (EHG) of 83 participants. Of them, 70 participants had normal progress of labor and delivered vaginally, while the remaining 13 participants had arrested/prolonged progress of labor and delivered through cesarean section. The EHG data was recorded for about 30 minutes or at least for about 6 to 7 contractions. Seven noninvasive wireless surface electrodes were used to record the uterine activity. The electrode placement on the abdomen is shown in fig.1 and is described in detail in [1].

## Detection of contractions

Identifying the start and endpoints of a contraction from the EHG recordings were challenging. Two methods, a filtering method [1] and a statistical method [1] described in the literature, were evaluated on EHG recordings of 30 participants. The EHG signals of these 30 participants summed up to around 700 minutes of recording. The data was recorded at around 3-4 cm cervical dilatation, and the normal range of contractions at this stage is 2-5 contractions per 10 minutes. Hence, the 700 minutes of recording is expected to contain around 140-350 contractions in total.

However, the filtering method was able to detect only 21 contractions in total from the 700 minutes of data. In the statistical method, the scaling factor (an arbitrarily chosen constant) of 2 used to define the threshold was too high for our data, and no contractions were detected. Hence, we reduced the scaling factor to 1.2 and were able to detect 65 contractions in total, which although better than the filtering method, it is still less than expected.

In order to better detect the contractions we employed the Teager Kaizer Energy Operator (TKEO), a popular method to detect contractions from EHG signals [17, 18, 19, 20, 21]. Mathematically it is expressed as in (i).


(i) $$y_{t}=x_{t}^{2}-\left\{\left(x_{t-i}\right)\left(x_{t+i}\right)\right\}$$

where, y_t_ = output sample, x = input sample, and t = time or sample number. In order to enhance the distinction between activity and noise, the z-score is calculated. It is mathematically expressed as in (ii).


(ii) $$z_{\text{score }}=\left(y_{t}-\mu \left(y_{tnc}\right)\right)/\left(\sigma \left(y_{tnc}\right)\right)$$

where, y = output sample, μ = mean, σ = standard deviation, and t_nc_ = time period where contraction activity is not present. The algorithm developed based on TKEO to identify the contractions is described in table 1.

**Table 1:** TKEO based algorithm to identify contractions.

TKEO	based algorithm to identify contractions
1.	The unprocessed EHG signals are obtained.
2.	A running mean (an averaging filter) filter is applied to suppress the short-term noise.
3.	Linear trends or baseline wandering (if observed) is eliminated by detrending the signal.
4.	TKEO and z-score is obtained for the detrended signal.
5.	A Gaussian-smoothing filter is applied to smoothen the signal.
6.	An envelope of the filtered signal is obtained.
7.	Contractions identified.

A threshold-based technique is used to segment the contractions identified from the TKEO process. The procedure to define a dynamic threshold based on the range of the values is described in [1], the same technique was used for our data. The algorithm to define the threshold is depicted in table 2.

**Table 2:** Algorithm to define threshold to segment contractions identified from the TKEO method.

Defining threshold to segment contractions from TKEO method	
1.	Obtain the contraction wave from the EHG signals using the TKEO process.
2.	A four-minute window of the RMS signal is chosen.
3.	Hanning window function is used to eliminate the edge effects.
4.	Set Threshold = 1.2*(basal tone + 25% signal range) where Basal tone = mean of 10% of the lowest values.
5.	If the sample value > threshold and is true for > 10 seconds, then
6.	It is identified as a contraction
7.	Else
8.	Move to next sample till the last sample in the four-minute window
9.	Slide the four-minute window by one minute & repeat steps 3 to 8

The identified regions were manually verified by the investigators based on a visual inspection and corrected if required, then segmented out. This approach yielded 192 contractions in total from 30 participants, which is found to be in the normal expected range.

Hence, the TKEO method was chosen for further analysis of the data of the remaining 53 participants. In total, 331 contractions were identified from the data of 70 participants in the normal progress group, and 53 contractions were identified from the data of 13 participants in the arrested/prolonged progress group.

**Fig. 2:**
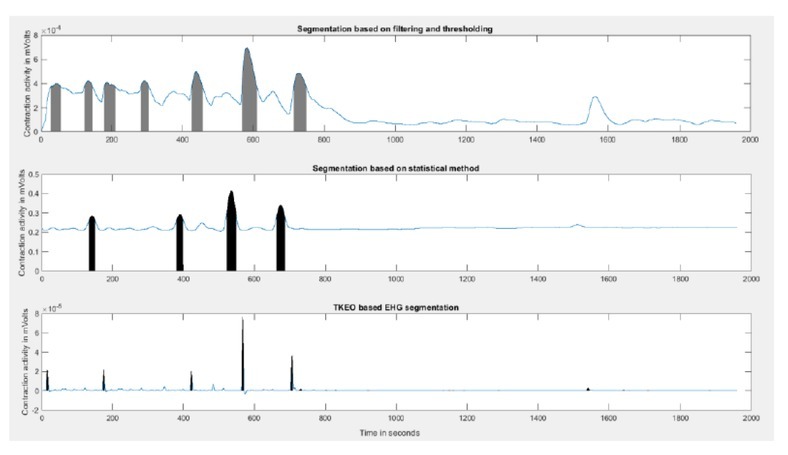
A comparison of contractions detected using the three methods for the same signal. Top represents the filtering method. Middle represents the statistical method. Bottom represents the TKEO method.

## Correlation analysis

The cross-correlation between two contractions is a great way to analyze the consistency of the contractions. For labor to progress normally, the contractions need to progressively increase both in terms of intensity and duration over the course of labor. However, for the short 30 mins of contractile activity that we have collected, it is safe to consider that they need to be consistent in terms of the force being applied on the uterine muscle. For each patient, the first contraction recorded was considered as the reference. Subsequently, each contraction was used to obtain the correlation coefficient against the reference contraction. The correlation coefficient represents the amount of similarity between the contractions. All the correlation coefficients obtained for each patient were normalized in order to be comparable amongst participants.

Five equal states were defined arbitrarily, ranging from 0 to 1, with an interval of 0.2, state 1 being the lowest (00.2), and state 5 is the highest (0.8-1). The contractions were categorized into these states based on the value of their correlation coefficients. The pattern of transition from one state to another by consecutive contractions were obtained. The transition probabilities were obtained for all 331 contractions combined in the normal progress group and all 53 contractions combined in the arrested/prolonged progress group.

## Dominant region determination

The role of fundal dominance in the normal progress of labor has been debated for long and results have been confounding [1], [1], [22, 23, 24, 25]. We investigated the dominance of electrical activity along the vertical axis of the umbilicus, in the upper uterine segment and the lower uterine segment. For this calculation alone, the EHG data was processed in a differential configuration. The differential signals from electrodes 1 & 2 gave the localized activity of the upper uterine segment and the differential signals from the electrodes 5 & 7 gave the localized activity of the lower uterine segment. The data from the other channels were ignored for this calculation. The time coordinates of contraction regions in a particular EHG signal obtained from the TKEO method were used to segment out unprocessed EHG signals belonging to the contraction portions of the signals. The bipolar signals were obtained from these unprocessed EHG signals.

**Fig. 3:**
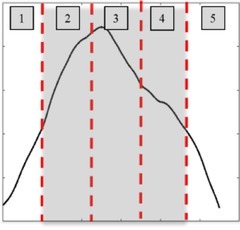
Five equi-temporal regions of a contraction

Each contraction region was divided into five equi-temporal regions, as shown in Fig.3, and the first and the last regions were neglected in the calculations owing to the low intensity of contractions. The RMS of the upper bipolar and the lower bipolar regions were calculated for each region, i.e., regions 2 to 4. The dominance was calculated as the difference between the upper uterine segment RMS and the lower uterine segment RMS. The algorithm is depicted in table 3.

**Table 3:** The algorithm to calculate the dominant region of the contraction.

1.	Bipolar signals BPU and BPL representing the electrical activity specific to the upper and lower uterine segments are calculated.
2.	An envelope of the BPU and BPL is obtained by using Hilbert’s transform.
3.	The entire duration of contraction is divided into five equal parts 1 to 5.
4.	The RMS amplitude is calculated for the upper and the lower bipolar signals for regions 2,3 and 4 (RMS-u2, RMS-u3, RMS-u4 and RMS-l2, RMS-l3, RMS-l4) representing the most substantial part of the contraction.
5.	Dominance is calculated for segments 2, 3 & 4 (D2, D3, D4).
6.	The upper uterine segment is dominant if D is positive, and the lower uterine segment is dominant if D is negative.
7.	The dominance for the entire contraction is considered to be the most common pattern of dominance in segments 2, 3, and 4.
8.	End

## Transition probabilities

For each particular patient, the first contraction recorded was considered the reference and subsequently, evaluated each contraction against the reference contraction using the cross-correlation function. The correlation coefficient represents the amount of similarity between the contractions. The correlation coefficients for each patient were normalized to range the values from 0 to 1.

The values between 0 and 1 were divided into five regions with an interval of 0.2 and categorized as the states. For example, if the normalized correlation coefficient of a contraction was 0.45, it was considered in state 2. The pattern of transition from one state to another by consecutive contractions was obtained and plotted these patterns of all patients combined as the transition probabilities as below.

**Table 4:** Transition probabilities of normal progress group*_._*

States	State 1	State 2	State 3	State 4	State 5
State 1	0.692	0.263	0.400	0.500	0.385
State 2	0.051	0.368	0.200	0.500	0.231
State 3	0.026	0.053	0.200	0.000	0.231
State 4	0.051	0.105	0.000	0.000	0.154
State 5	0.179	0.211	0.200	0.000	0.000

**Table 5:** Transition probabilities of arrested/prolonged progress group

States	State 1	State 2	State 3	State 4	State 5
State 1	0.667	0.333	1.000	0.000	0.667
State 2	0.067	0.000	0.000	0.000	0.000
State 3	0.067	0.000	0.000	0.000	0.000
State 4	0.000	0.000	0.000	1.000	0.333
State 5	0.200	0.667	0.000	0.000	0.000

## Informed consent

Informed consent has been obtained from all individuals included in this study.

## Ethical approval

The research related to human use has been complied with all relevant national regulations, institutional policies and in accordance with the tenets of the Helsinki Declaration and has been approved by the authors’ institutional review board or equivalent committee.

## Results

The TKEO method to detect contractions worked the best for our data compared to the filtering method and the statistical method. Hence, this method was chosen for analysis of all the 83 participants. In total, 331 contractions were identified from 70 participants who delivered vaginally, and 53 contractions were identified from the 13 participants who faced prolonged/arrested progress of labor.

**Fig. 4:**
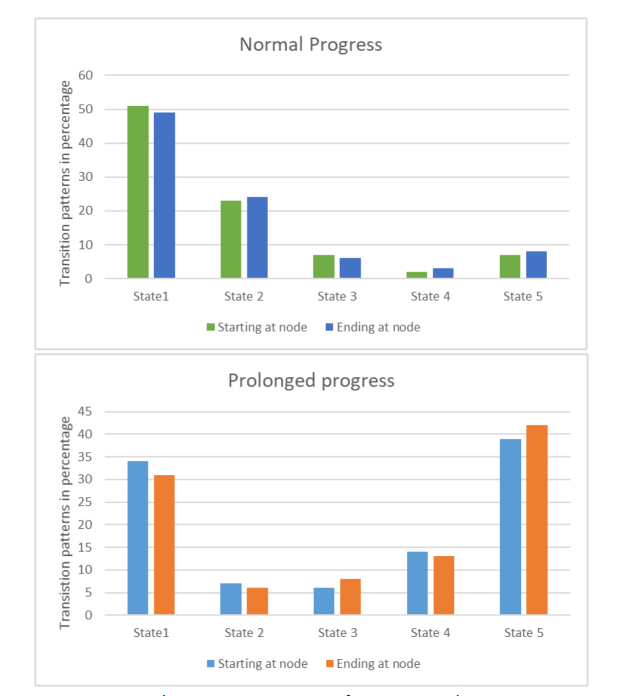
The transition patterns of contractions between the states represented in percentage

These transition patterns are represented in Figure 4. In the normal progress cases, the good correlation between the contractions is evident in the way that the transitions are concentrated among the states 1 and 2. On the contrary, in the prolonged progress cases, the poor correlation between the contractions is seen as the transitions are the highest in the state 5. In other words, the consistency of contractions is better in the normal progress group compared to the arrested/prolonged progress.

The dominant regions of the uterus during regions 2 to 4 of a contraction is represented in table 6. There is a noticeable difference in the dominant regions of the uterine segments. In prolonged progress cases, around three-fourth of the contractions were dominant in the upper uterine segment, whereas in the normal progress group, around three-fifths of the contractions were dominant in the lower uterine segment.

**Table 6:** Dominant region of the uterus during contractions.

Feature	Group	Upper uterine segment	Lower uterine segment
	Normal progress	38.94%	61.05%
Dominance	arrested Prolonged/ progress	73.58%	26.41%

## Markov Chain

**Fig. 5:**
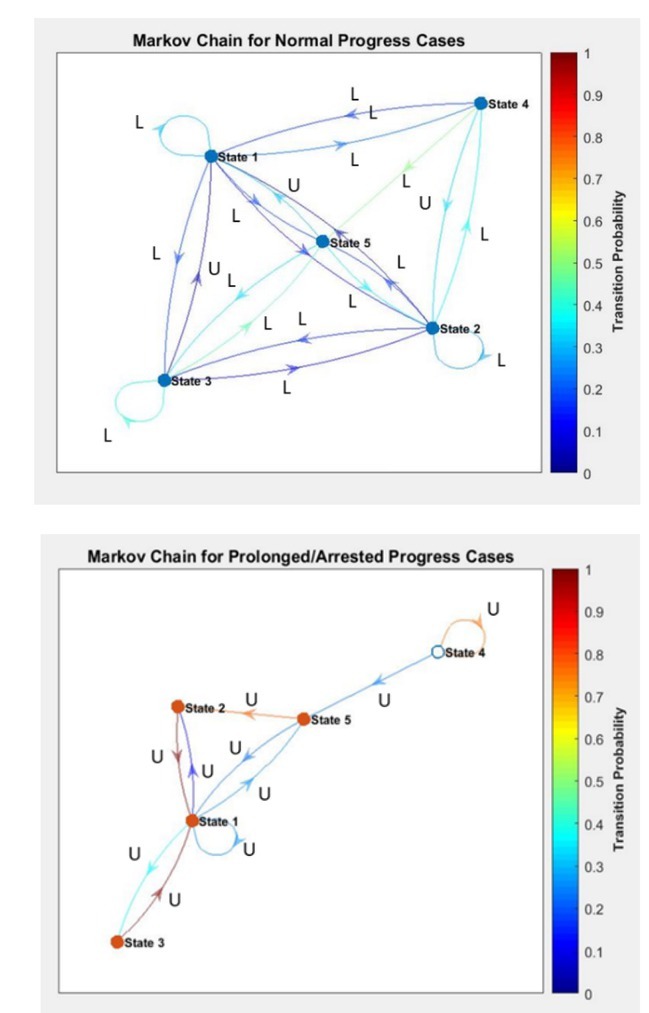
Representation of the Markov chain correlation coefficients and the dominance of the uterine segment associated with them.

For the labor to progress normally, the contractions need to be consistent in terms of the force being applied on the uterine muscle. We used the correlation coefficient to evaluate the consistency of the contractions. In this exercise, 80% (306 contractions) of the EHG data was used to develop the model and the remaining 20% (77 contractions) of the data was used to validate the model. The most common pattern of dominance and its associated correlation coefficients have been represented as a Markov Chain in Figure 5. The dominance of each contraction was mapped to the corresponding transition states. In Figure 5, the ‘U’ signifies that the upper uterine segment is dominant, the ‘L’ the lower uterine segment. The most repetitive dominant region is represented as the common dominant region for a particular transition state.

Based on these observations, we defined a simple threshold-based rule to predict the risk of the prolonged labor. The rule considers both the consistency of the contractions as well as the dominant uterine segment during the contractions. The threshold for the consistency for the labor to be prolonged more than 50% of the transitions should be in either state 4 or 5. The threshold for dominant uterine segment for the labor to be prolonged more than 50% of the contractions should be dominant in the upper uterine segment. In case, only one threshold is met, the weightage must be given for the dominant region of uterine segment based solely on the reason that it is a binary classification (i.e., upper or lower) as compared to a 5-stage classification in the consistency of contractions, hence likely to be more reliable in a short duration of EHG recording.

**Fig. 6:**
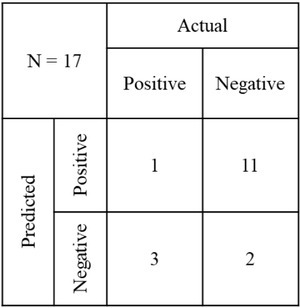
Confusion matrix of the prediction of the prolonged labor for the test data.

Based on the thresholds defined above we tested the prediction accuracy on the remaining 20% of the data. The accuracy of prediction was 82.35%, with a sensitivity of 75% and specificity 33.33%. The confusion matrix for this is represented in Figure 6.

## Discussion

Consistency of the contractions could be a potential indicator of the progress of labor. This, coupled with the dominant region of the uterus during contractions, could identify the risk of prolonged/arrested progress of labor. The patterns observed occur at the start of the active labor, i.e., 3–4 cm of cervical dilatation, thus making it inherently predictive in nature. This means the model predicts the outcomes 2 to 4 hours prior to the delivery or the requirement of intervention.

This early prediction provides ample time to shift the patient to a higher care center in case of low resource settings. And in higher care centers, this performs the role of a triaging tool. Thus, improving the treatment planning, resource and staffing optimization, and efficiency of the hospital. But most importantly, this would help in identifying the cases requiring an intervention, in turn, reducing the mortality, and morbidities associated with dystocia. While at the same time, it would also reduce the unnecessary cesarean sections again reducing the mortality, morbidity and the expenditure related to it.
